# Clinical outcomes of surgical resection versus radiofrequency ablation in very-early‐stage hepatocellular carcinoma: a propensity score matching analysis

**DOI:** 10.1186/s12876-021-01995-z

**Published:** 2021-11-08

**Authors:** Yuan-Chen Li, Ping-Hung Chen, Jen-Hao Yeh, Pojen Hsiao, Gin-Ho Lo, TaoQian Tan, Pin-Nan Cheng, Hung-Yu Lin, Yaw-Sen Chen, Kun-Chou Hsieh, Pei-Min Hsieh, Chih-Wen Lin

**Affiliations:** 1 School of Medicine, College of Medicine, I-Shou University, Kaohsiung, Taiwan; 2 Division of Gastroenterology and Hepatology, Department of Medicine, E-Da Hospital, I-Shou University, No. 1, Yida Road, Jiaosu Village, Yanchao District, Kaohsiung City, 82445 Taiwan; 3 Division of Gastroenterology and Hepatology, I-Shou University, E-Da Dachang Hospital, Kaohsiung, Taiwan; 4 Health Examination Center, E-Da Hospital, I-Shou University, Kaohsiung, Taiwan; 5 Department of Surgery, E-Da Hospital, I-Shou University, Kaohsiung, Taiwan; 6 School of Chinese Medicine, College of Chinese Medicine, China Medical University, Taichung, Taiwan; 7 Research Center for Traditional Chinese Medicine, China Medical University Hospital, Taichung, Taiwan; 8 Division of Gastroenterology and Hepatology, Department of Internal Medicine, College of Medicine, National Cheng Kung University Hospital, National Cheng Kung University, Tainan, Taiwan

**Keywords:** Surgical procedures, Radiofrequency ablation, Very-early-stage hepatocellular carcinoma, Overall survival, Disease-free survival

## Abstract

**Background:**

The detection rate of Barcelona Clinic Liver Cancer (BCLC) very-early-stage hepatocellular carcinoma (HCC) is increasing because of advances in surveillance and improved imaging technologies for high-risk populations. Surgical resection (SR) and radiofrequency ablation (RFA) are both first‐line treatments for very-early-stage HCC, but the differences in clinical outcomes between patients treated with SR and RFA remain unclear. This study investigated the prognosis of SR and RFA for very-early‐stage HCC patients with long‐term follow‐up.

**Methods:**

This study was retrospectively collected data on the clinicopathological characteristics, overall survival (OS), and disease-free survival (DFS) of 188 very-early-stage HCC patients (≤ 2 cm single HCC). OS and DFS were analyzed using the Kaplan–Meier method and Cox regression analysis. Propensity score matching (PSM) analysis was performed.

**Results:**

Of the 188 HCC patients, 103 received SR and 85 received RFA. The median follow‐up time was 56 months. The SR group had significantly higher OS than the RFA group (10-year cumulative OS: 55.2% and 31.3% in the SR and RFA groups, respectively). No statistically significant difference was observed in DFS between the SR and RFA groups (10-year cumulative DFS: 45.9% and 32.6% in the SR and RFA groups, respectively). After PSM, the OS in the SR group remained significantly higher than that in the RFA group (10-year cumulative OS: 54.7% and 42.2% in the SR and RFA groups, respectively). No significant difference was observed in DFS between the SR and RFA groups (10-year cumulative DFS: 43.0% and 35.4% in the SR and RFA groups, respectively). Furthermore, in the multivariate Cox regression analysis, treatment type (hazard ratio (HR): 0.54, 95% confidence interval (CI): 0.31–0.95; *P* = 0.032) and total bilirubin (HR: 1.92; 95% CI: 1.09–3.41; *P* = 0.025) were highly associated with OS. In addition, age (HR: 2.14, 95% CI: 1.36–3.36; *P* = 0.001) and cirrhosis (HR: 1.79; 95% CI: 1.11–2.89; *P* = 0.018) were strongly associated with DFS.

**Conclusion:**

For patients with very-early-stage HCC, SR was associated with significantly higher OS rates than RFA. However, no significant difference was observed in DFS between the SR and RFA groups.

## Introduction

Hepatocellular carcinoma (HCC) is the fifth most common malignancy worldwide, and the third most common cause of cancer-related deaths [1]. Patients with Barcelona Clinic Liver Cancer (BCLC) very-early-stage HCC (BCLC stage 0) have well-preserved liver function with a single tumor less than 2 cm [2–4]. The detection rate of very-early-stage HCC is increasing because of advances in surveillance technology for at-risk populations [5–7]. According to the American Association for the Study of Liver Disease (AASLD), the European Association for the Study of Liver (EASL), and the Asian Pacific Association for the Study of the Liver (APASL) guidelines, the currently recommended treatment options for very-early‐stage HCC are surgical resection (SR), radiofrequency ablation (RFA), and liver transplantation [2–4]. Because of the organ shortage, SR and RFA are the most common treatments for very-early-stage HCC patients [8, 9].

It is still debated whether SR or RFA is more effective for treating very-early-stage HCC. Many previous studies have demonstrated that RFA is as effective for treating small liver tumors as SR [10–18]. Another study reported that RFA may be the first choice for patients with BCLC stage 0 HCC [19]. However, recent studies have demonstrated that SR is associated with higher overall survival (OS) and disease‐free survival (DFS) rates compared with RFA in HCC patients with a single small HCC [12, 20–25]. Conflicting information still arises because of the lack of long-term survival and recurrence rate data. Therefore, we designed a retrospective study to compare the long-term survival and recurrence rates associated with SR and RFA. Furthermore, propensity score matching was used to minimize the effects of confounding factors.

## Methods

**Fig. 1:**
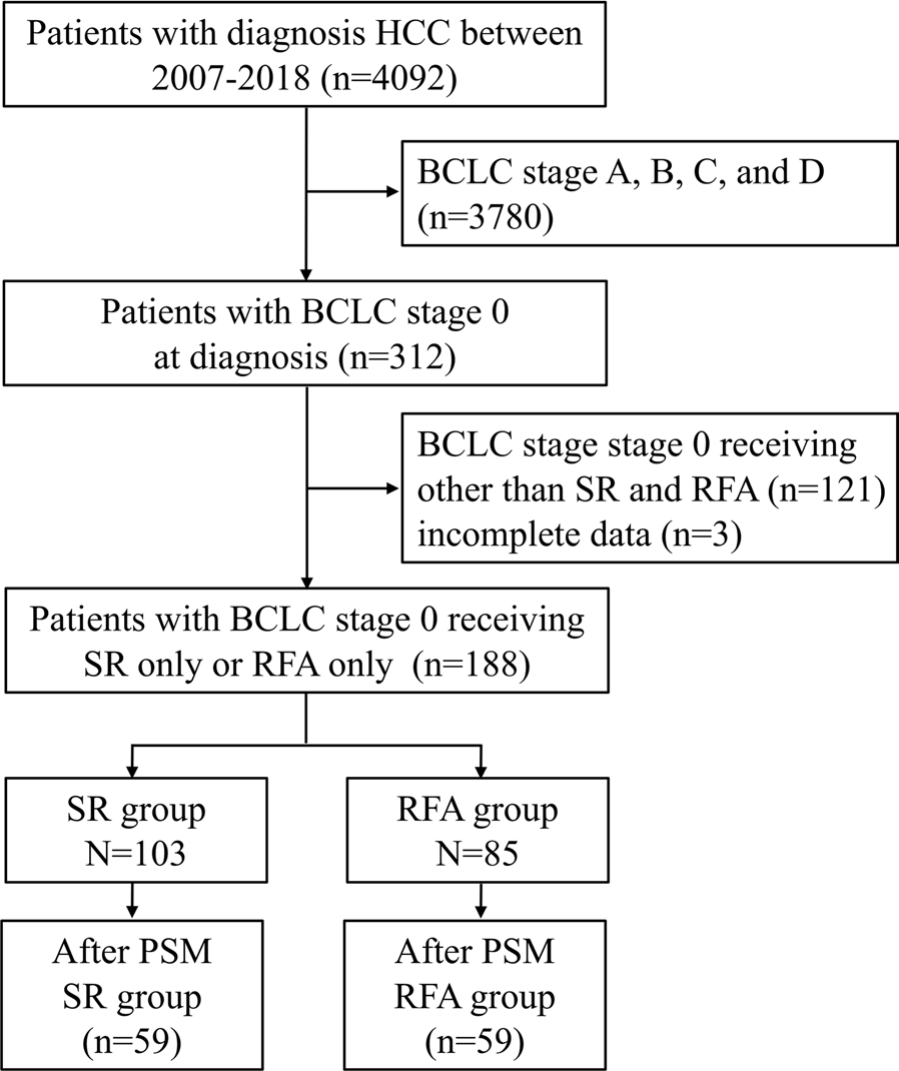
Study flowchart and inclusion of participants

Data from 4092 HCC patients at E-Da Hospital, I-Shou University, Kaohsiung, Taiwan from 2007 to 2018 were retrospectively collected. However, 3780 patients were excluded because they had BCLC stage A, B, C, and D HCC, and 124 patients were excluded because they were receiving treatments other than SR and RFA or had incomplete data. Therefore, this prospective study enrolled 188 HCC patients who received only SR or RFA treatment (Fig. 1). Our study was approved by the Institutional Review Board of E-Da Hospital. HCC diagnosis was based on the criteria of the practice guidelines of the EASL or AASLD [2, 3].

Clinicopathological parameters included the following demographic data: sex and age, body mass index (BMI), excessive alcohol use, infection with hepatitis B virus (HBV) infection and hepatitis C virus (HCV), tumor size, liver function, mortality, recurrence, and follow-up time. Tumor size and liver cirrhosis were diagnosed and evaluated through histopathologic and radiological findings, namely computed tomography (CT) or magnetic resonance imaging (MRI) findings with non-specific gadolinium and EOB-MRI. The liver function data were primarily measured using a hematology test and assessed using Child–Pugh (CP) scores.

### HCC treatment

Patients were treated with SR or RFA; our multidisciplinary team selected a suitable therapy after considering the patient’s preference and the medical evidence. Patients who received SR were evaluated according to the subsequent remnant liver volume, with consideration of tumor‐free resection margins and hepatic functional reserves [26]. The RFA endpoint was the complete ablation of both the visible tumor and tumor margins. Patients were followed up every 3 to 6 months with abdominal ultrasound and CT or MRI, and alpha-fetoprotein (AFP) levels were assessed. OS was defined as the time from the date of diagnosis to the date of death or last visit. DFS was defined as the time from the date of diagnosis to the date of HCC recurrence or last visit. The last follow-up date was in August 2018.

### Data analysis and statistics

All statistical analyses were performed using SPSS ver. 23.0 (SPSS, Chicago, IL, USA). Numerical data are expressed as medians and ranges, and categorical data are described using numbers and percentages. OS and DFS were determined using the Kaplan–Meier method and compared between patients receiving different treatments. Cox proportional hazards regression analysis of OS and DFS was also performed. Furthermore, we used logistic regression to generate propensity score matching (PSM) with age, sex, tumor size, cirrhosis, total bilirubin, albumin, and AFP level to reduce bias in our analyses. The two treatment groups were matched with the control group according to PSM using a caliper width of 0.02. After PSM, the baseline covariates were compared using the paired *t* test or Mann–Whitney U test for continuous variables and the chi-square test for categorical variables. A *p* value of < 0.05 indicated statistical significance.

## Results

### Patient characteristics

The demographic data of patients with BCLC very-early-stage HCC are summarized in Table 1. Of the 188 patients, 103 received SR and 85 received RFA. The median follow-up time was 56 months (range: 6–142 months). Patients were significantly younger in the SR group than in the RFA group. The liver function, including total bilirubin, albumin, international normalized ratio (INR), and albumin–bilirubin (ALBI) grade, was lower in the RFA group. In the entire cohort, 45.7% of patients were HBV positive, and 48.4% were HCV positive. No significant difference was observed in the number of HBV-positive and HCV-positive patients between the SR group and the RFA group. The rate of cirrhosis was significantly higher in the RFA group than in the SR group.

**Table 1:** Clinical characteristics of BCLC very-early stage hepatocellular carcinoma patients before and after propensity scores matching

Characteristics	Study population before PSM				Study population after PSM			
	Total	SR group	RFA group	*P-*value	Total	SR group	RFA group	*P-*value
Patients	188	103	85		116	58	58	
Age, years	59 (23–82)	57 (23–82)	62 (34–81)	0.006	61 (34–82)	61 (35–82)	61 (34–80)	0.788
*Sex*								
Male	130(69.1)	75 (72.8)	55 (64.7)	0.299	78 (67.2)	39 (67.2)	39 (67.2)	1
Female	58 (30.9)	28 (27.2)	30 (35.3)		38 (32.8)	19 (32.8)	19 (32.8)	
*BMI*								
≦ 24 kg/m^2^	93 (49.5)	50 (48.5)	43 (50.6)	0.895	59 (50.9)	29 (50.0)	30 (51.7)	1
> 24 kg/m^2^	95 (50.5)	53 (51.5)	42 (49.4)		57 (49.1)	29 (50.0)	28 (48.3)	
Alcohol use	53 (28.2)	25 (24.33)	28 (32.9)	0.249	32 (27.6)	15 (25.9)	17 (29.3)	0.835
*HBV*								
Positive	86 (45.7)	51 (49.5)	35 (41.2)	0.320	51 (44.0)	28 (48.3)	23 (39.7)	0.454
Negative	102 (54.3)	52 (50.5)	50 (58.8)		65 (56.0)	30 (51.7)	35 (60.3)	
*HCV*								
Positive	91(48.4)	49 (47.6)	42 (49.4)	0.917	61 (52.6)	34 (58.6)	27 (46.6)	0.265
Negative	97 (51.6)	54 (52.4)	43 (50.6)		55 (47.4)	24 (41.4)	31 (53.4)	
Child‐Pugh class A	179 (95.2)	100 (97.1)	79 (92.9)	0.326	113 (97.4)	56 (96.6)	57(98.3)	1
Cirrhosis	115 (61.2)	54 (52.4)	61 (71.8)	0.011	72 (62.1)	36 (62.1)	36 (62.1)	1
Ascites	13 (6.9)	6 (5.8)	7 (8.2)	0.719	8 (6.9)	4 (6.9)	4 (6.9)	1
INR	1.0 (0.9–1.7)	1.0 (0.9–1.3)	1.1 (0.9–1.7)	< 0.001	1.0 (0.9–1.7)	1.0 (0.9–1.3)	1.0 (0.9–1.7)	0.382
Total bilirubin, mg/dL	0.9 (0.2–5.4)	0.8 (0.2–5.4)	1.1 (0.3–4.2)	0.001	1.0 (0.3–3.9)	0.8 (0.5–3.9)	1.1 (0.3–4.2)	0.785
Albumin, g/dL	4.1 (2.5–4.9)	4.2 (3.2–4.9)	4.0 (2.5–4.7)	< 0.001	4.1 (2.6–4.9)	4.1 (3.2–4.9)	4.0 (2.6–4.6)	0.051
ALBI grade 1	166 (88.3)	97 (94.2)	69 (81.2)	0.011	102 (87.9)	53 (91.4)	49 (84.5)	0.393
AFP > 200 ng/ml	29 (15.4)	12 (11.7)	17 (20.0)	0.169	17 (14.7)	5 (8.6)	12 (20.7)	0.115
Tumor size, cm	1.8 (1.0–2.0)	1.8 (1.0–2.0)	1.8 (1.0–2.0)	0.127	1.8(1.0–2.0)	1.9 (1.0–2.0)	1.8 (1.0–2.0)	0.875
Mortality	69 (36.7)	28 (27.2)	41 (48.2)	0.005	44 (37.9)	17 (29.3)	27 (46.6)	0.085
Recurrence	79 (42.0)	43 (41.7)	36 (42.4)	1.000	49 (42.2)	25 (43.1)	24 (41.4)	1.000

Data shown as median (range) or number (%); PSM: propensity score matching; SR: Surgical resection; RFA: Radiofrequency ablation; BMI: Body mass index; HBV: Hepatitis B virus; HCV: Hepatitis C virus; INR: international normalized ratio; ALBI: Albumin- bilirubin; AFP: Alpha-fetoprotein;

### Overall survival and disease-free survival before propensity score matching

**Fig. 2:**
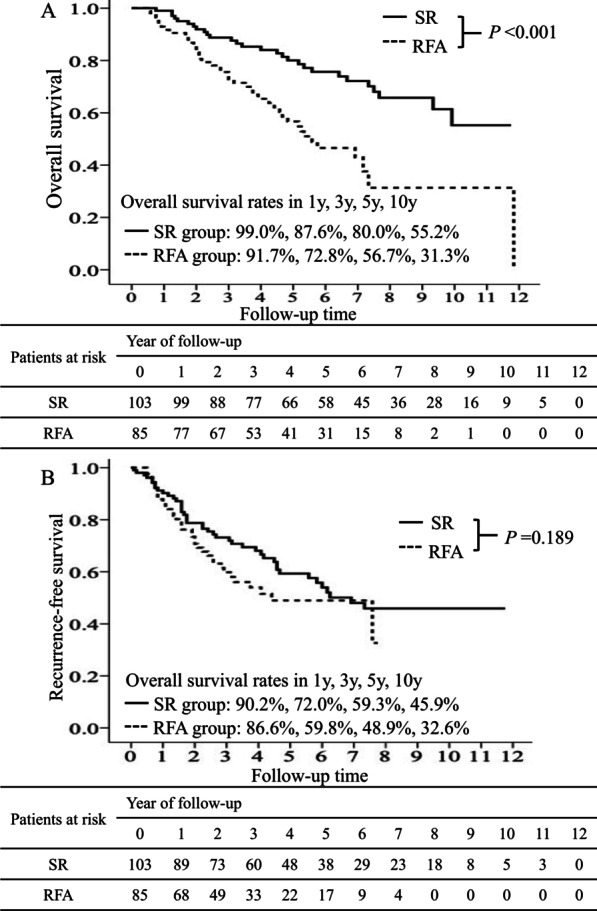
Overall survival and disease-free survival for different treatments in patients with Barcelona Clinic Liver Cancer stage 0 hepatocellular carcinoma. The cumulative incidence of overall survival (**A**) and disease-free survival (**B**) for different treatments. Surgical resection (SR) resulted in significantly higher overall survival rates than radiofrequency ablation (RFA) (*P* < 0.001) (**A**). No significant difference was observed in disease-free survival between SR and RFA (**B**)

In total, 28 and 41 patients died in the SR group and the RFA group, respectively. OS was significantly higher in the SR group than in the RFA group (*P* < 0.001) (Fig. 2A). The 1-, 3-, 5-, and 10-year cumulative OS rates were 99.0%, 87.6%, 80.0%, and 55.2% in the SR group and 91.7%, 72.8%, 56.7%, and 31.3% in the RFA group, respectively. In addition, 43 and 36 patients experienced HCC recurrence in the SR and RFA groups, respectively. No statistically significant difference in DFS was observed between the SR and RFA groups (Fig. 2b). The 1-, 3-, 5-, and 10-year cumulative DFS rates were 90.2%, 72.0%, 59.3%, and 45.9% in the SR group and 86.6%, 59.8%, 49.8%, and 32.6% in the RFA group, respectively.

### Overall survival and disease-free survival after propensity score matching

**Fig. 3:**
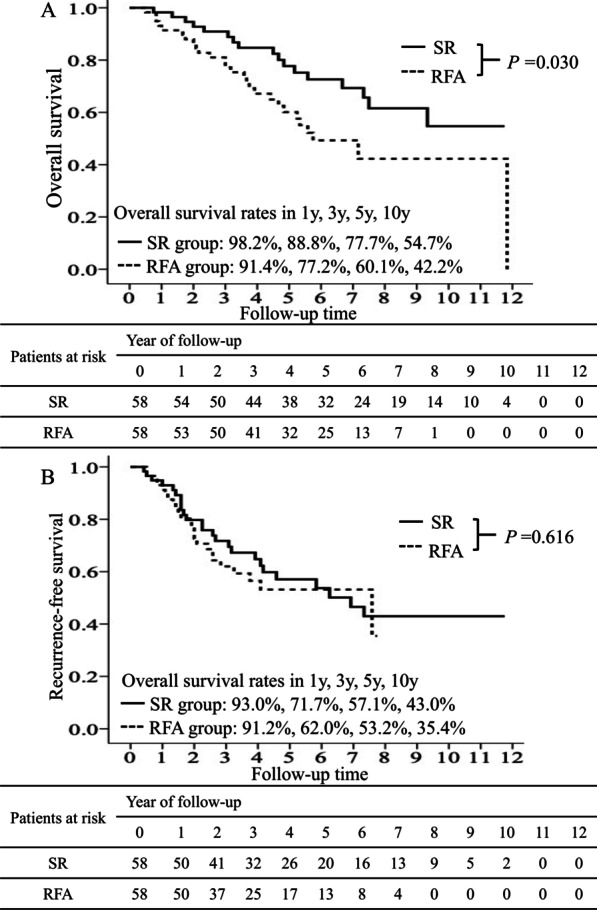
Overall survival and disease-free survival for different treatments after propensity score matching. The cumulative incidence of overall survival (**A**) and disease-free survival (**B**) for different treatments after propensity score matching. Overall survival remained significantly higher in surgical resection (SR) than in radiofrequency ablation (RFA) (*P* < 0.001) (**A**). No significant difference was observed in disease-free survival between SR and RFA (**B**)

After PSM, the total number of patients was 116, with 58 patients in the SR group and 58 patients in the RFA group. None of the clinical features were significantly different between the SR and RFA groups (Table 1). A total of 18 and 27 patients died in the SR and RFA groups, respectively. OS was significantly higher in the SR group than in the RFA group (*P* = 0.03) (Fig. 3a). The 1-, 3-, 5-, and 10-year cumulative OS rates were 98.2%, 88.8%, 77.7%, and 54.7% in the SR group and 91.4%, 77.2%, 60.1%, and 42.2% in the RFA group, respectively. Furthermore, 25 and 24 patients experienced HCC recurrence in the SR and RFA groups, respectively. No statistically significant difference in DFS was observed between the SR and RFA groups (Fig. 3b). The 1-, 3-, 5-, and 10-year cumulative DFS rates were 93.0%, 71.7%, 57.1%, and 43.0% in the SR group and 91.2%, 62.0%, 53.2%, and 35.4% in the RFA group, respectively.

### Prognostic factors associated with overall survival and disease-free survival

**Fig. 4:**
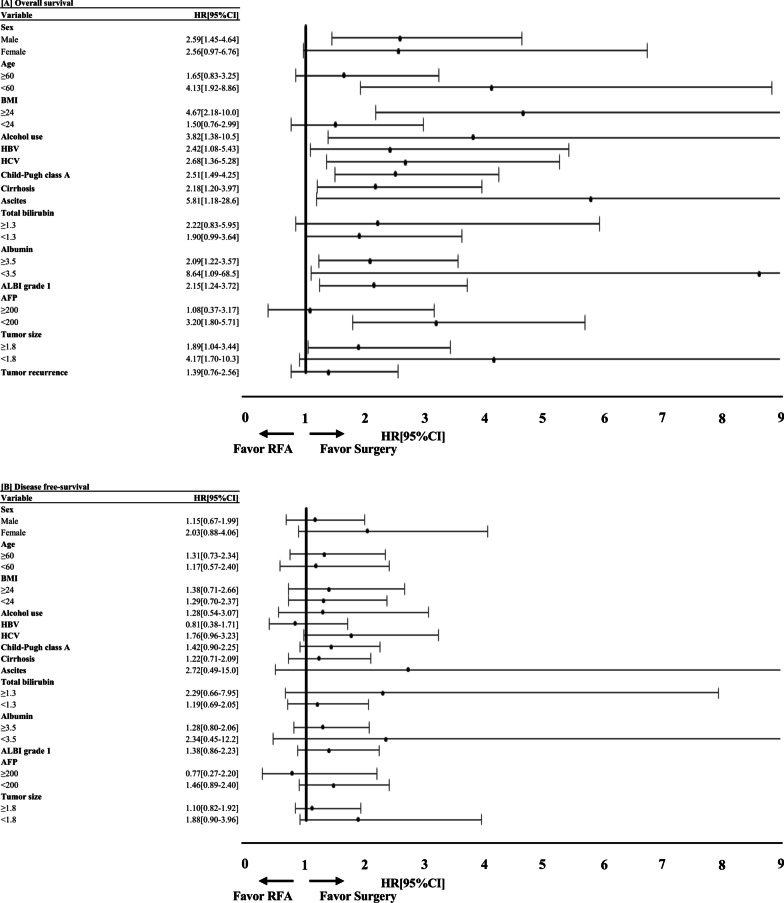
Forest plot of the treatment effect on overall survival and recurrence‐free survival in subgroup analyses. The effect of surgical resection (SR) was better overall survival than radiofrequency ablation (RFA) in the almost subgroups (**A**). The effect of SR was similar DFS with RFA in all subgroups (**B**)

Treatment type, CP class A, cirrhosis, ascites, serum total bilirubin, serum albumin, ALBI grade, and serum AFP were strongly associated with OS in the univariate analysis (Table 2). The multivariate Cox regression analysis revealed that the treatment type (hazard ratio (HR): 1.75, 95% confidence interval (CI): 1.08–3.11; *P* = 0.046), serum total bilirubin (HR: 0.52; 95% CI: 0.28–0.95; *P* = 0.034), and tumor recurrence (HR: 0.46; 95% CI: 0.28–0.78; *P* = 0.006) were strongly associated with OS (Table 2). The effect of SR was better OS than RFA in the almost subgroups (Fig. 4a).

**Table 2:** Univariable and multivariable Cox‐proportional hazard model for overall survival in BCLC very-early stage hepatocellular carcinoma patients

Characteristics	Univariate Cox regression analyses		Multivariate Cox regression analyses	
	Unadjusted HR (95% CI)	*P*-value	Adjusted HR (95% CI)	*P-*value
Treatment type (SR vs. RFA)	2.58 (1.56–4.25)	< 0.001	1.75 (1.08–3.11)	0.046
Male	1.20 (0.71–2.04)	0.501		
Age (≥ 60y vs, < 60y)	0.74 (0.46–1.19)	0.219		
BMI (≥ 24 kg/m^2^ vs. < 24 kg/m^2^)	1.06 (0.66–1.71)	0.808		
Alcohol use	0.71 (0.43–1.19)	0.196		
HBV	1.58 (0.96–2.59)	0.073		
HCV	0.74 (0.46–1.19)	0.212		
Child‐Pugh class A	2.60 (1.19–5.70)	0.017	1.21 (0.46–3.17)	0.697
Cirrhosis	0.52 (0.31–0.88)	0.015	0.90 (0.50–1.60)	0.709
Ascites	0.39 (0.20–0.76)	0.006	0.56 (0.28–1.13)	0.104
Total bilirubin (≥ 1.3 mg/dL vs. < 1.3 mg/dL)	0.33 (0.20–0.54)	< 0.001	0.52 (0.28–0.95)	0.034
Albumin (≥ 3.5 g/dL vs. < 3.5 g/dL)	2.55 (1.33–4.87)	0.005	1.59 (0.74–3.38)	0.232
ALBI grade 1 vs. 2/3	1.91 (1.04–3.52)	0.036	1.10 (0.52–2.35)	0.798
AFP (≥ 200 ng/ml vs. < 200 ng/ml)	0.56 (0.31–1.00)	0.051		
Tumor size (≥ 1.8 cm vs. < 1.8 cm)	0.68 (0.41–1.13)	0.137		
Tumor recurrence	0.45 (0.28–0.74)	0.002	0.46 (0.28–0.78)	0.006

CI: Conference Incidence; SR: Surgical resection; BMI: Body mass index; HBV: Hepatitis B virus; HCV: Hepatitis C virus; ALBI: Albumin- bilirubin; AFP: Alpha-fetoprotein;

Age and cirrhosis were strongly associated with DFS in the univariate analysis (Table 3). The multivariate Cox regression analysis revealed that age (HR: 0.47, 95% CI: 0.30–0.73; *P* = 0.001) and cirrhosis (HR: 0.56; 95% CI: 0.35–0.90; *P* = 0.018) were strongly associated with DFS (Table 3). The effect of SR was similar DFS with RFA in all subgroups (Fig. 4b).

**Table 3:** Univariable and multivariable Cox‐proportional hazard model for disease-free survival in BCLC very-early stage hepatocellular carcinoma patients

Characteristics	Univariate Cox regression analyses		Multivariate Cox regression analyses	
	Unadjusted HR (95% CI)	*P*-value	Adjusted HR (95% CI)	*P-*value
Treatment type (SR vs. RFA)	1.35 (0.86–2.11)	0.193		
Male	0.97 (0.60–1.56)	0.903		
Age (≥ 60y vs, < 60y)	0.49 (0.31–0.76)	0.002	0.47 (0.30–0.73)	0.001
BMI (≥ 24 kg/m^2^ vs. < 24 kg/m^2^)	1.29 (0.83–2.02)	0.256		
Alcohol use	1.04 (0.63–1.72)	0.871		
HBV	1.29 (0.83–2.03)	0.256		
HCV	0.73 (0.47–1.14)	0.164		
Child‐Pugh class A	1.14 (0.42–3.13)	0.795		
Cirrhosis	0.59 (0.37–0.96)	0.032	0.56 (0.35–0.90)	0.018
Ascites	0.76 (0.35–1.65)	0.482		
Total bilirubin (≥ 1.3 mg/dL vs. < 1.3 mg/dL)	0.87 (0.52–1.47)	0.609		
Albumin (≥ 3.5 g/dL vs. < 3.5 g/dL)	1.08 (0.50–2.36)	0.840		
ALBI grade 1 vs. 2/3	0.82 (0.39–1.70)	0.589		
AFP (≥ 200 ng/ml vs. < 200 ng/ml)	0.78 (0.44–1.40)	0.406		
Tumor size (≥ 1.8 cm vs. < 1.8 cm)	1.19 (0.75–1.87)	0.467		

CI: Conference Incidence; SR: Surgical resection; BMI: Body mass index; HBV: Hepatitis B virus; HCV: Hepatitis C virus; ALBI: Albumin- bilirubin; AFP: Alpha-fetoprotein

## Discussion

In our study, SR provided better OS than RFA, both before and after propensity score matching. In patients with BCLC very-early-stage HCC, the 10-year cumulative OS rates were 55.2% and 31.3% in the SR and RFA groups, respectively (*P* < 0.001). Furthermore, no significant differences in DFS were observed between the SR and RFA groups. The 10-year cumulative OS rates were 45.9% and 32.6% in the SR and RFA group, respectively. The risk factors identified for OS were the treatment type and tumor size, and the risk factors identified for DFS were age and cirrhosis.

Our study also demonstrated that the SR group had higher OS than the RFA group, whereas no significant difference in DFS was observed between the SR and RFA groups. These results are different from those of several previous studies, which have reported that patients with small HCC who received SR had higher OS and DFS than those who received RFA [20–25]. Furthermore, our study results conflict with those from a previous study showing that SR resulted in higher DFS, but not OS, compared with RFA [12]. By contrast, our study observed that RFA resulted in similar DFS but lower OS compared with SR. Our results were also different from a previous study that reported higher OS and DFS for RFA than for SR. Many studies have demonstrated that OS and DFS are similar for patients treated with RFA and SR [10, 11, 13–17, 22, 27, 28]. In addition, our results were different from those of a previous study showing that RFA resulted in higher OS and similar DFS compared with SR [19].

Our study showed that RFA, lower liver function (i.e., lower serum albumin, higher bilirubin, and lower ALBI grade), and the presence of cirrhosis or ascites were significantly associated with lower OS rates according to the univariate analysis. These factors have been identified by previous studies [18, 29]. Furthermore, older age and the presence of cirrhosis were significantly associated with lower DFS rates, which supports the results of a multicenter Italian survey [27]. However, a nationwide cohort in Japan demonstrated that older patients may receive RFA rather than SR because of the presence of comorbidities [30]. Therefore, the treatment type may impact the results of recurrence. Although patients receiving RFA in our study were older than those receiving SR, we did not identify an association between treatment type and recurrence rates.

Previous studies showed that median post-recurrence OS after SR was 26 months [31] and median post-recurrence OS after RFA was 22 months [32]. Our study demonstrated that median post-recurrence OS after SR and RFA was 34 and 32 months, respectively. Our results were better OS post-recurrence after SR and RFA treatment. Furthermore, there is currently no standard of care for adjuvant therapy in HCC after curative treatments because evidence is limited in HCC patients after potentially curative treatment [33–35].

Conventional transcatheter arterial chemoembolization (cTACE) was an optional treatment for HCC and doxorubicin-loaded drug-eluting beads TACE (DEB-TACE) has been developed to maximize the therapeutic efficacy of cTACE for HCC. DEB-TACE was more cost-effective than conventional TACE when a minimum willingness-to-pay was accepted, mainly depending on shorter in-hospital stay and better quality of life [36]. Moreover, it is an important issue to explore the economic impact between SR and RFA treatment in very-early stage HCC. RFA had similar life-expectancy and quality-adjusted life-expectancy at a lower cost than SR and was the most cost-effective therapeutic strategy for very-early stage HCC patients in a 10-year perspective [22]. However, SR remains the best strategy to adopt as a result of better OS at an acceptable increase in cost.

This study has several limitations. First, it is a retrospective study and is thus highly vulnerable to potential bias; even after propensity score matching, some confounding factors are unavoidable. The best solution to mitigate this bias is to conduct a randomized controlled trial. However, randomization is difficult because the treatment decision must consider the patients’ physical conditions, presenting ethical concerns. Second, the relatively low number of patients may lead to type I error, which influences the results of univariate analyses. The limited number of certain events also made it difficult to perform robust multivariate analyses. Therefore, the results of the multivariate analyses require validation in a cohort with a larger sample size and a greater number of events.

## Conclusions

SR was associated with a superior OS rate than RFA. However, no significant difference was observed in the DFS rate between the SR and RFA groups. The risk factors identified for OS were treatment type and serum total bilirubin, and the risk factors for DFS were age and cirrhosis.

## Data Availability

Data is available from the corresponding author upon reasonable request.

## Abbreviations

HCC: Hepatocellular carcinoma
BCLC: Barcelona Clinic Liver Cancer
AASLD: American Association for the Study of Liver Disease
ESAS: European Association for the Study of Liver
APASL: Asian Pacific Association for the Study of the Liver
SR: Surgical resection
RFA: Radiofrequency ablation
OS: Overall survival
DFS: Disease-free survival
CP score: Child–Pugh score
HBV: Hepatitis B virus
HCV: Hepatitis C virus
PSM: Propensity score matching
HR: Hazard ratio
CI: Confidence interval
ECOG: Eastern Cooperative Oncology Group
